# Touching moments: desire modulates the neural anticipation of active romantic caress

**DOI:** 10.3389/fnbeh.2014.00060

**Published:** 2014-02-28

**Authors:** Sjoerd J. Ebisch, Francesca Ferri, Vittorio Gallese

**Affiliations:** ^1^Department of Neuroscience and Imaging, G. d’Annunzio UniversityChieti, Italy; ^2^Institute of Advanced Biomedical Technologies (ITAB), G. d’Annunzio UniversityChieti, Italy; ^3^Department of Neuroscience, Section of Physiology, Parma UniversityParma, Italy; ^4^Mind, Brain Imaging and Neuroethics, University of Ottawa Institute of Mental Health ResearchOttawa, ON, Canada

**Keywords:** affective touch, social touch, active touch, desire, reward anticipation, posterior insula, fMRI, affilliative behavior

## Abstract

A romantic caress is a basic expression of affiliative behavior and a primary reinforcer. Given its inherent affective valence, its performance also would imply the prediction of reward values. For example, touching a person for whom one has strong passionate feelings likely is motivated by a strong desire for physical contact and associated with the anticipation of hedonic experiences. The present study aims at investigating how the anticipatory neural processes of active romantic caress are modulated by the intensity of the desire for affective contact as reflected by passionate feelings for the other. Functional magnetic resonance imaging scanning was performed in romantically involved partners using a paradigm that allowed to isolate the specific anticipatory representations of active romantic caress, compared with control caress, while testing for the relationship between neural activity and measures of feelings of passionate love for the other. The results demonstrated that right posterior insula activity in anticipation of romantic caress significantly co-varied with the intensity of desire for union with the other. This effect was independent of the sensory-affective properties of the performed touch, like its pleasantness. Furthermore, functional connectivity analysis showed that the same posterior insula cluster interacted with brain regions related to sensory-motor functions as well as to the processing and anticipation of reward. The findings provide insight on the neural substrate mediating between the desire for and the performance of romantic caress. In particular, we propose that anticipatory activity patterns in posterior insula may modulate subsequent sensory-affective processing of skin-to-skin contact.

## Introduction

A romantic caress is a primary expression of affiliative behavior. It reflects the disposition of individuals to seek close contact between them, and it promotes socio-emotional relationships, pair bonding and reproduction (Dunbar, 2010; Gallace and Spence, 2010; Morrison et al., 2010). Despite the interactive character of tactile interactions among humans, they are addressed almost exclusively as a receptive experience by psychological and neuroscientific investigations. Moreover, active romantic caress essentially has a socio-emotional intention through somatosensory interaction with another individual. Hence, its anticipatory neural processes already could be uniquely modulated according to sensory-motor, emotional and social factors. Indeed, during action, predictions are made by the brain about its consequences in order to motivate initiation, anticipate effects and optimize performance (Wolpert et al., 1995; Blakemore et al., 1998; Knoblich and Flach, 2001; Schutz-Bosbach and Prinz, 2007; Haggard, 2008). Elucidating the anticipatory neural mechanisms underlying active romantic caress will add elementary information to the understanding of how social interaction is driven by brain function, motivating and coordinating behavior.

In addition to its sensory-motor component, romantic caress is inherently associated with an affective component (Hertenstein et al., 2006; Gallace and Spence, 2010; Morrison et al., 2010). Regarding the anticipation of passive tactile experiences, sensory and affective consequences are coded by brain circuits related to sensory-motor and affective processes (Porro et al., 2002; Lovero et al., 2009; Gazzola et al., 2012; Morrison et al., 2013). Behavioral evidence suggests that the prediction of outcomes of performed actions also comprises an affective component that modulates the perception of action consequences (Wilke et al., 2012). Furthermore, touch is a primary reinforcer strongly connected with motivational and affective processes that may drive social behavior (Rolls, 2010), and some studies demonstrated that rewards influenced the processing of tactile stimuli (Pleger et al., 2008, 2009). Thus, active romantic caress could be anticipated by neural processes associated with emotions, motivating social behavior and modulating effects of touch performance. However, romantic caress is a rather intimate domain where its affective evaluation is subjective and depends on many factors (Fisher et al., 1976; DiBiase and Gunnoe, 2004). A powerful factor influencing this affective component is the relationship between the interacting individuals. For example, Gazzola et al. (2012) showed that, when manipulating the perceived affective quality (pleasantness) of a passively experienced caress, primary somatosensory cortex (SI) encoded, and was modulated by, the perceived gender of the caresser. Likewise, touching a person for whom one has strong feelings of passionate love could be associated with hedonic experiences and motivated by a strong desire for physical contact (Hatfield and Sprecher, 1986; Gallace and Spence, 2010).

Whereas previous studies started to elucidate the neural basis of affective, social touch experiences, it remains poorly understood whether and how anticipatory neural processes of active romantic caress performance vary as a function of the desire for affective contact with the other. Co-variance of anticipatory neural processes with passionate feelings for the other could indicate the anticipation of hedonic experiences and prediction of reward due to tactile interactions. The present study aims at investigating how the anticipatory neural processes of an active romantic caress are modulated by the desire for affective contact as reflected by the intensity of feelings of passionate love for the other. An additional issue concerns the question whether anticipatory neural activity patterns associated with desire are independent of perceptual affective qualities, like pleasantness. Although the desire for union with another likely is related to pleasantness, it also could comprise more abstract representations of love and affection, partly associated with distinct neural substrates (Fisher et al., 2002; Cacioppo et al., 2012). Alternatively, anticipatory processes associated with the desire for romantic caress could correspond to those for anticipating the perceived affective qualities of touch.

For this purpose, functional magnetic resonance imaging (fMRI) scanning was performed in romantically involved partners using a paradigm that allowed to isolate the specific anticipatory representations of romantic caress, compared with a control caress. Main statistical analysis tested for the relationship between neural activity anticipating active romantic caress, compared to control caress, and measures of feelings of passionate love for the other. It was hypothesized that brain regions known to be associated with the affective valence of experienced romantic caress as well as reward processing and prediction also contribute to the anticipation of active romantic caress, subsequently modulating their response to actual experience.

A particular role is expected for posterior insula in relationship with feelings of desire for union with the other. Posterior insula is generally involved in processing somatosensory stimuli with affective or motivational significance, and is considered a central brain region for interoception, whereas, along with the integration of cognitive and emotional responses, anterior insula underpins the subjective awareness of bodily feeling states (Craig, 2002, 2009). Based on these functions, posterior insula has been proposed to generate predictions about bodily feeling states (Paulus and Stein, 2006) and to provide a crucial neural substrate for reward-processing (Paulus, 2007). Especially relevant in the context of pleasant skin-to-skin contact (Morrison et al., 2010), coding of pleasant romantic caress has been associated with posterior insula (Morrison et al., 2011) based on the C-tactile fiber system projecting to posterior insula (Olausson et al., 2002; Löken et al., 2009). Insular cortices also have been related to sexual desire and love with a posterior-to-anterior pattern, suggesting that desire and love are on a spectrum that evolves from integrative representations of affective visceral sensations (Cacioppo et al., 2012). Moreover, an fMRI study combined with selective serotonin reuptake inhibitors administration provided further evidence for anticipatory processing of emotional stimuli in posterior insula (Simmons et al., 2009). Thus, posterior insula could have a peculiar role in the anticipation of active tactile contact with others mediating between the desire for and the experience of performing a romantic caress.

## Materials and methods

### Participants

Twenty-four healthy, right-handed, young adults (age 19–34 years; 12 female) participated in the present experiment. In particular, the participant group was composed of 12 heterosexual, romantically involved couples (relationship >2 months, <5 years) that were recruited by means of advertisements on the university campus. Exclusion criteria for all participants included physical health problems and neurological hard signs, standard contraindications for fMRI, a history of severe head trauma, loss of consciousness, drug abuse. During scanning, romantic caress was investigated within the couples by scanning both partners in randomized order. The study was approved by the local Ethics Committee. Written informed consent was obtained from all participants after full explanation of the procedure of the study, in line with the Declaration of Helsinki.

### functional magnetic resonance imaging (fMRI) data acquisition

For each subject, Blood Oxygen Level Dependent (BOLD) contrast functional imaging was performed with a General Electric scanner at Parma University at 3 T by T2*-weighted gradient echo-planar sequences with the following parameters: *TR* = 2000 ms, *TE* = 30 ms, matrix size 64 × 64, field of view (*FoV*) = 20.5 mm, in-plane voxel size = 3.2031 × 3.2031 mm, flip angle = 90°, slice thickness = 3 mm, and a 0.5 mm gap. A 8HRBRAIN head coil was used. Functional volumes consisted of 42 transaxial slices that were acquired in a sequential descending order. A high-resolution structural volume was acquired at the end of the session.

### Experimental procedure and materials

Four fMRI runs (256 functional volumes/run) were acquired for each participant. The participant was in a supine position in the fMRI scanner. A wooden table was placed on the participant’s legs. The participant’s right hand was placed in the center of the table with an object (a ball that was fixed on the table) and the hand of her/his partner (who was standing next to the scanner) both placed next to the participant’s hand.

The partner’s hand represented the target of the romantic caress condition, whereas the ball represented the target of the control caress condition. An object (ball) was chosen as a control target due to the necessity to minimize emotional involvement in the control condition as much as possible, while keeping the way of touching (i.e., caressing) constant. Indeed, social touch in general is inherently associated with an affective component and often rather intimate (e.g., Fisher et al., 1976; DiBiase and Gunnoe, 2004; Hertenstein et al., 2006; Gallace and Spence, 2010; Morrison et al., 2010). This makes it difficult to obtain an emotionally neutral condition, even when caressing a non-partner’s or a stranger’s hand. Given that the aim was to study how brain activity anticipating an active romantic caress, relative to a control caress, co-varies with individual ratings of passionate love for the other, it is very unlikely that such a relationship can be explained by differences induced by an animate vs. an inanimate target.

**Figure 1:**
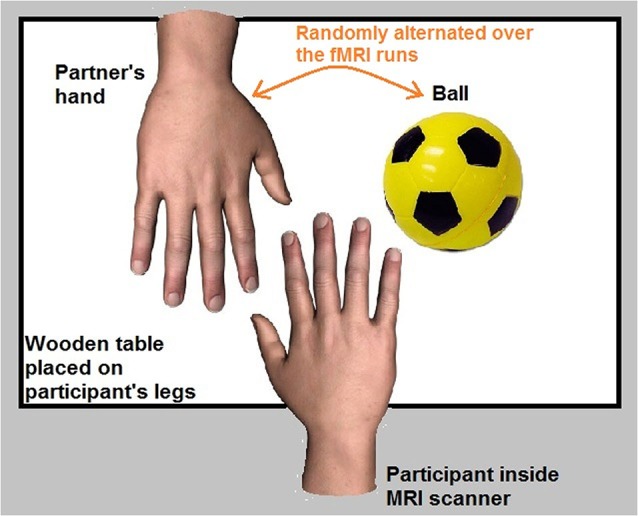
**Experimental setup**.

To avoid systematic effects of the location where the partner’s hand and the object were placed, the position of the partner’s hand and the ball was pseudorandomized throughout the experiment (i.e., on the right and left side of the participant’s hand). Before each individual fMRI run, participants were informed about on which side of their own hand the partner’s hand and the ball were placed. Behavioral performance accuracy of participants was monitored during the experiment through a video camera placed in the MRI room. The experimental setup is illustrated in Figure 1.

During fMRI scanning, participants completed a series of touch and no touch trials. Each trial, either touch or no touch, started with a visual cue consisting of a black and white line outline drawing of either a hand or a circle. The visual cues were presented for a duration of 1000 ms and were always followed by a red fixation cross. After 3000 ms, the red fixation cross could become either green (duration = 6000 ms) or black (variable duration = 4000/6000/8000 ms).

In case the red fixation cross became green (33% of the trials), participants were required to pleasantly caress the partner’s hand or the object in the same way, according to the cue. These trials were defined as “touch trials”. If the preceding cue was a hand, participants had to caress the back of the hand of their partner (romantic caress performance, 28 trials). If the preceding cue was a circle, participants had to caress the ball (control condition, 28 trials). When the green fixation cross turned black, participants had to bring their hand in the original position on the table.

In case the red fixation cross became black (67% of the trials), participants had to keep their hand on the table and to wait for the next cue. These trials were defined as “no touch trials”. Because the touch trials occurred randomly, participants could not know beforehand whether they had to perform the cued touch. Therefore, participants were required to prepare either a partner- or object direct caress in all the trials (i.e., “touch trials” as well as “no touch trials”). The “no touch” trials were of principal interest for data analysis, since they reflected the touch intention in anticipation of touch performance, without the presence of any overt movements of the participant. Thus, the touch intention could be to romantically caress (56 trials; romantic caress intention) or to perform a control caress (56 trials; control caress intention).

Prior to scanning, participants underwent a practicing session outside the scanner in order to train them on the fMRI task. With respect to the touch performance trials, participants were trained to caress either the hand or the object over a surface of 6 cm with a velocity of 3 cm/s (Morrison et al., 2011).

**Figure 2:**
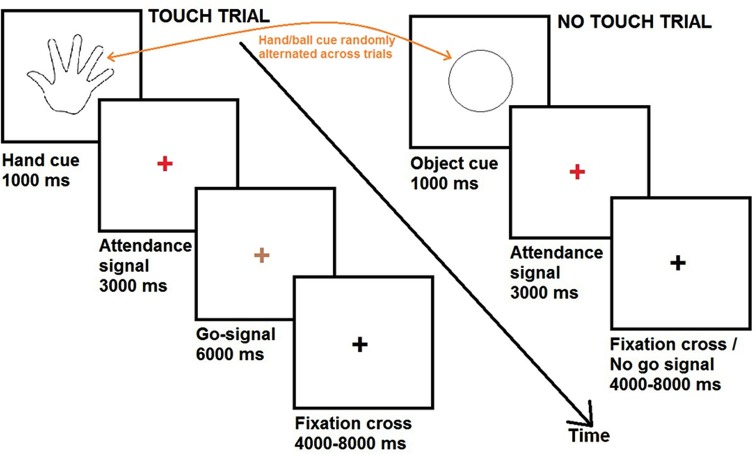
**Time course of the experiment**.

The experimental procedure is visualized in Figure 2.

### Debriefing

At debriefing outside the scanner room all participants completed the Passionate Love Scale (PLS; Hatfield and Sprecher, 1986), a self-report questionnaire assessing the intensity of feelings of passionate love. Passionate love is here defined as a state of intense longing for union with the other, mentally, emotionally and physically. The questionnaire consists of 15 statements involving cognitive, emotional and behavioral components. Participants are required to provide an answer to every statement on a scale from 1 (not at all true) to 9 (definitely true). Participants were instructed to think about the person with whom they performed the experiment when responding on the items of the questionnaire and to respond as honestly as possible. They were assured that the results were strictly confidential and that their partner would not have been informed about their responses.

Furthermore, participants were asked to rate the pleasantness of the experience of touching their partner or the object during scanning. For this purpose, participants indicated the subjectively perceived pleasantness of touch experiences with a pencil on a Visual Analogue Scale (VAS) consisting of a 10 cm vertical line where the lowest point meant very unpleasant and the highest point very pleasant.

Participants were not informed about the content of the ratings prior to scanning to avoid potential effects of attention directed to particular aspects of the experiment or experiences.

### functional magnetic resonance imaging (fMRI) data preprocessing and analysis

Raw data were analyzed with the Brain Voyager QX 2.3 software (Brain Innovation, Maastricht, The Netherlands). Due to T1 saturation effects, the first five scans of each run were discarded from the analysis. Pre-processing of functional data included slice scan time correction, motion correction and removal of linear trends from voxel time series. A three-dimensional motion correction was performed with a rigid-body transformation to match each functional volume to the reference volume estimating three translation and three rotation parameters. Pre-processed functional volumes of a participant were co-registered with the corresponding structural data set. As the 2D functional and 3D structural measurements were acquired in the same session, the co-registration transformation was determined using the slice position parameters of the functional images and the position parameters of the structural volume.

Structural and functional volumes were transformed into the Talairach space (Talairach and Tournoux, 1988) using a piecewise affine and continuous transformation. Functional volumes were resampled at a voxel size of 3 × 3 × 3 mm and spatially smoothed with a Gaussian kernel of 6 mm full-width half maximum to account for intersubject variability. The fMRI runs were modeled by means of a two gamma hemodynamic response function using predictors for the different no touch conditions (cue, red cross) and the different touch conditions (cue, red cross; green cross). The intertrial interval (black cross) was not included as a predictor and, hence, not modeled as a separate predictor, but used as baseline period (rest).

Prior to statistical analysis, a percent signal change normalization of the time series from the different runs was performed as implemented as a default function in Brain Voyager QX software. This scaling allows to normalize a voxel’s time course in such a way that the mean signal value will be transformed to a value of 100 and the individual values will be fluctuating around that mean as percent signal deviations. Normalized fMRI responses indicate differences across conditions regardless of the variability in the fMRI signal across subjects, scanning sessions, and voxels. The parameters (beta values) estimated in individual subject analysis were entered in a second level voxel-wise random effect group analysis in order to search for activated areas that were consistent for the whole group of participants. In order to obtain a significance level corrected for multiple comparisons, the uncorrected *p*-value (*p* < 0.001) of the statistical maps and an estimate of the spatial correlation of voxels were used as input in a Monte Carlo simulation (10000 simulations) to access the overall significance level and to determine a cluster size threshold (*k*) associated with a corrected value of *p* < 0.01 (Forman et al., 1995).

Statistical analyses of task-evoked BOLD-responses to the “no touch trials” were divided in four steps:

1. Statistical maps related to the “no touch trials” (contrasts: romantic caress anticipation vs. baseline; control caress anticipation vs. baseline) were calculated by means of voxel-wise, whole-brain *t*-tests. This analysis allowed to detect BOLD-responses, compared with baseline, independently for the two conditions.
2. To test whether there were differences in BOLD-responses between the anticipation of a romantic caress and the anticipation of a control caress, direct contrasts were performed between the “no touch trials” of these two conditions by means of a voxel-wise, whole-brain *t*-test.
3. In order to investigate the relationship between BOLD responses to the “no touch” conditions (i.e., touch anticipation) and the intensity of the feelings of passionate love between the participating partners, covariance analyses were performed. Also this analysis concerned a whole-brain, voxel-wise approach. Beta-values resulting from the contrast between the romantic caress anticipation condition and the control caress anticipation condition were set as dependent variable, and PLS scores as covariate.
4. In addition to the voxel-wise analysis described in the previous steps, a control region of interest (ROI)-based analysis was performed. The purpose of this additional step was to test for the specificity of covariance effects for the intensity of passionate feelings, controlling for effects due to pleasantness. Partial correlation analysis was performed with beta-values within the ROI as obtained by the voxel-wise co-variance analysis described in step 3. For this analysis, the average beta-value resulting from the contrast between the romantic caress intention condition and the control caress intention condition was extracted from all voxels within the ROI. Pleasantness ratings concerned the difference between the pleasantness of the romantic caress performance and the control caress performance as rated by the participants. Thus, partial correlation coefficients were calculated taking into account the relative contribution of both PLS scores and pleasantness ratings to explain variance among individual beta-values (romantic caress vs. control caress).

Additional analysis was performed on the “touch trials”. It needs to be mentioned that this analysis was rather preliminary, for example, due to the small number of touch trials and variability in touch performance. Indeed, the touch trials primarily served as catch trials. In this case, the phase were the green cross was present was analyzed, i.e., when participants were actually performing the touch, excluding the preceding cue and red cross phases on the trial.

An explorative ROI analysis according to a random effect model was performed in order to test with greater specificity whether brain regions that showed a relationship between neural activity during the “no touch” trials and the intensity of passionate feelings showed a differential response to the romantic and control caress performance. For this analysis, beta values (i.e., based on the average time-series across the voxels within the clusters) concerning the “touch trials” were extracted from a priori ROIs, that is, the voxel clusters showing a significant effect regarding the covariance analysis on the “no touch trials”. Independent sample *t*-tests were performed to test for significant differences between the experimental conditions.

Finally, in order to preliminary test whether the brain regions involved in the anticipation of active caress also responded differently to actual caress performance, a whole-brain, voxel-wise conjunction analysis was performed. A random effect analysis of the conjunction between two contrasts was based on the minimum statistic compared to the conjunction null, controlling the false positive error for conjunction inference, and testing for common activations by creating the intersection of statistical maps thresholded at a specific alpha-rate (Nichols et al., 2005). Specifically, the following contrast was performed: [“romantic caress” vs. “control caress” no touch trials] ∩ [“romantic caress” vs. “control caress” touch trials]. Analysis of caress anticipation concerned the “no touch trials”, whereas the analysis of touch performance focused on the green cross phase of the “touch trials”.

### functional magnetic resonance imaging (fMRI) functional connectivity analysis

For functional connectivity analysis, a second step of data preprocessing was performed by using self-devised MATLAB (The Mathworks Inc., Natick, MA) scripts. These included: (1) bandpass filtering between 0.009 and 0.08 Hz (Cordes et al., 2001; Fox and Raichle, 2007; Auer, 2008); (2) regression of global, white matter, and ventricle signals, and their first derivatives (Fox et al., 2009; Van Dijk et al., 2010); (3) regression of three dimensional motion parameters, and their first derivatives; and (4) regression of task-related BOLD fluctuations (Fair et al., 2007; He et al., 2007; Van Dijk et al., 2010; Ebisch et al., 2013); scrubbing of motion affected functional volumes (Power et al., 2012).

Functional connectivity analysis was performed in terms of brain long-range communication identifying low-frequency, temporally-correlated patterns of continuous BOLD fluctuations across brain regions (Fox and Raichle, 2007; Van Dijk et al., 2010). This method allows a voxel-wise, whole-brain analysis that is not constrained to a pre-definite set of brain regions, as required by effective connectivity approaches, like dynamic causal modeling (Friston, 2011). Furthermore, rather than focusing on task-evoked BOLD-responses in task-specific brain regions as required by psychophysical interaction analysis (O’Reilly et al., 2012), it allows to test for functional interactions of task-common brain regions independent of BOLD-responses analyzed by conventional analyses of task-evoked neural activity.

Specifically, functional connectivity maps were calculated by means of voxel-wise, whole brain analyses, for brain ROIs showing a relationship between BOLD-responses anticipating active romantic caress and PLS scores obtained by the above described analysis. The ROIs were defined as a sphere with a 6 mm radius and were based on the peak coordinates of task-evoked activation patterns.

For all individual participants, we calculated correlations of BOLD fluctuations over continuous fMRI time-series between the seed ROI time-courses and the time-courses of all individual brain voxels. For this analysis, all four fMRI runs of each participant were concatenated. After applying Fisher’s *r*-to-*z* transformation (Zar, 1996) to each correlation map, random-effect group analysis was performed in order to reveal functional connectivity patterns that were consistent across participants. Statistical significance was determined by means of one-sample *t*-tests. Group statistical maps were thresholded at *p* < 0.0001 corrected for multiple comparisons by the False Discovery Rate (FDR; Genovese et al., 2002).

In addition, to investigate whether functional connectivity of the seed-ROIs varied as a function of desire for union with the other, a similar functional connectivity analysis were applied, but with PLS scores as covariate.

## Results

### Behavioral analysis

Mean score on the PLS for the total group of participants was 106 (standard deviation, *SD* = 10) on a scale ranging from 15 to 135. There was no significant difference in PLS score between male and female participants (*p* > 0.1).

On a scale ranging from 0 to 10, the mean of the pleasantness ratings was 8.9 (*SD* = 0.9) for the act of touching the partner and 5.4 (*SD* = 2.4) for the act of touching the object. A significant difference was found between the pleasantness of touching the partner or the object (*p* < 0.0001). There were no significant differences in pleasantness ratings between male and female participants (*p* > 0.1).

Correlation analysis failed to show a significant statistical dependency between PLS scores and pleasantness ratings (romantic vs. control caress performance) of the participants (*r* = 0.37, *p* = 0.08).

### functional magnetic resonance imaging (fMRI) data analysis: “no touch trials”

The romantic caress intention condition, compared with baseline, elicited significant activation in left supplementary motor area (SMA), left premotor cortex, bilateral posterior parietal cortex, bilateral extrastriate cortex, bilateral occipital cortex, bilateral anterior insula, bilateral fusiform gyrus (FG) (*p* < 0.01 corrected, *t* > 3.77, *k* > 8). Significant deactivations, compared with baseline, were found in bilateral primary and secondary somatosensory cortex (S2), bilateral posterior insula, bilateral superior parietal cortex (SPC), bilateral inferior parietal lobule, bilateral mid cingulate cortex (MCC), bilateral precentral gyrus (PreCG), right inferior frontal gyrus, right cerebellum (*p* < 0.01 corrected, *t* > 3.61, *k* > 8). The control caress intention condition, compared with baseline, elicited activation and deactivation patterns similar to the romantic caress (*p* < 0.01 corrected, *t* > 3.61, *k* > 8).

Regarding differences between the intention to romantically caress and the intention to perform a control caress, direct contrasts showed significantly differential BOLD-responses (*t* > 3.61, *p* < 0.01 corrected) in left postcentral sulcus (PostCS) and right primary somatosensory cortex (SI; Brodmann Area, BA, 2). In both regions, a stronger deactivation (negative BOLD modulation, compared to baseline) was observed in anticipation of a romantic caress, compared to a control caress.

**Figure 3:**
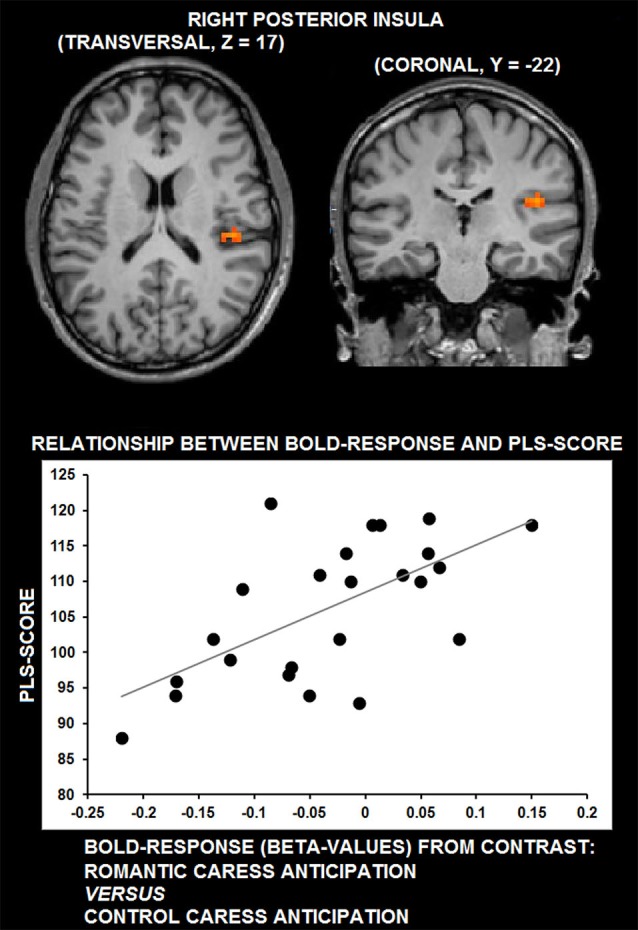
**Group statistical maps of right posterior insula activity covarying with PLS scores (thresholded at *p* < 0.01 corrected, corresponding to *r* > 0.63), and scatter plot depicting the correlation between anticipatory BOLD-response for romantic caress in posterior insula and PLS scores**.

Whole-brain, voxel-wise covariance analysis yielded a significant effect of PLS in right posterior insula (*r* > 0.63; *p* < 0.01 corrected). Specifically, the effect indicated a positive relationship between PLS scores, and the difference in neural activation during the romantic caress intention and the control caress intention condition. Thus, an increased response to the romantic caress intention condition, compared with the control caress intention condition, was associated with a higher PLS score. Statistical information about the co-variance analysis results is provided in Table 1. Group statistical maps and scatter plots regarding the covariance analysis are depicted in Figure 3.

**Table 1:** **Statistical details of activation foci obtained by group fMRI analysis**.

**Brain region**	**Talairach peak coordinate**	**Cluster size**	**Statistical value peak coordinate**	***p*-value peak coordinate**
*Analysis:*			
*Co-variance analysis (correlation BOLD-response romantic caress anticipation vs. control caress anticipation—PLS scores)*				
Right posterior insula	44, −23, 18	378	*r* = 0.69	<0.0005
*Analysis:*
*Conjunction analysis [romantic caress vs. control caress “no touch trials”] ∩ [romantic caress vs. control caress “touch trials”]*				
Left postcentral sulcus	−52, −26, 30	405	*t* = 3.987	<0.0005
Right primary somatosensory cortex	35, −29, 42	108	*t* = 3.987	<0.001

ROI-based control analysis using partial correlation analysis showed that statistical dependency between neural activity in right posterior insula during the “no touch” trials and PLS score was independent from pleasantness ratings; no significant correlation was detected between BOLD-response and pleasantness (*r* = 0.15, *p* = 0.48).

### functional magnetic resonance imaging (fMRI) data analysis: “touch trials”

**Figure 4:**
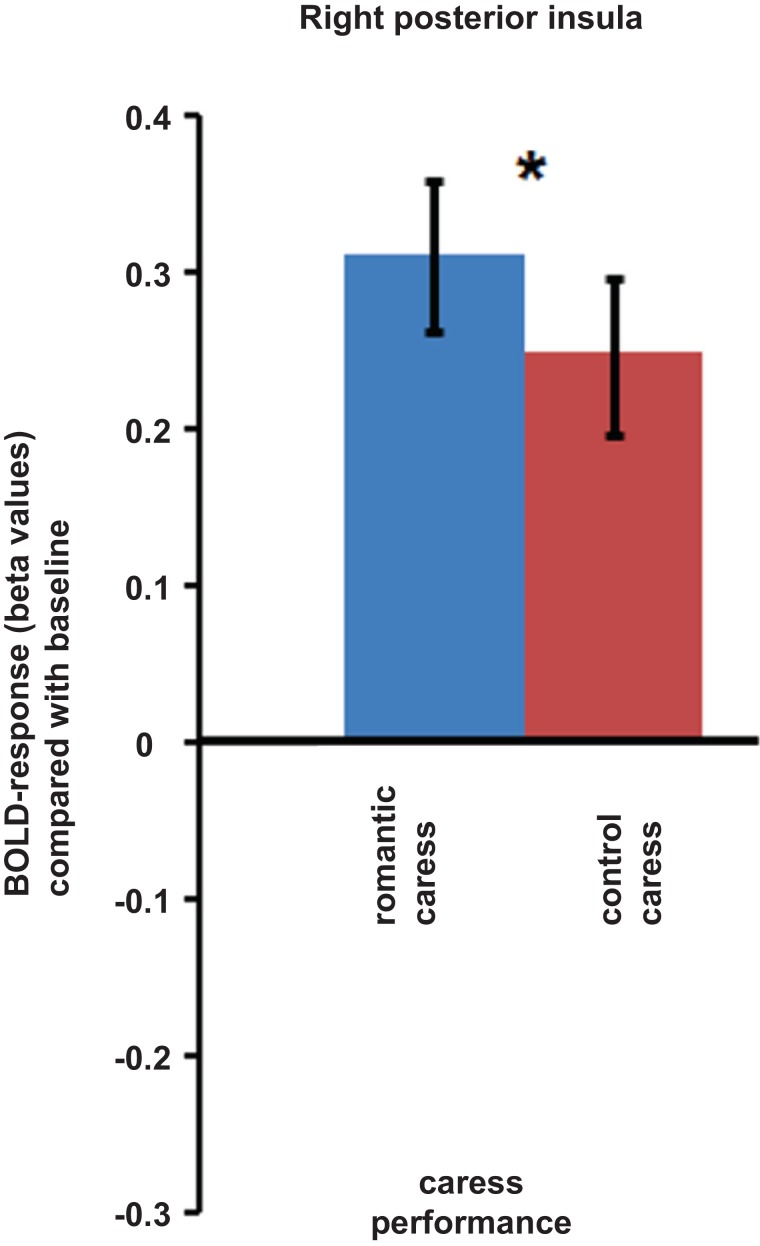
Graphs depicting BOLD-response in right posterior insula during the performance of romantic and control caress (* *p* < 0.05).

An ROI-based analysis assessing differential responses during the romantic caress and control caress performance in right posterior insula (i.e., the cluster showing a significant co-variance effect of PLS scores during the “no touch trials”) showed a significant modulation of BOLD-response by experimental condition during the “touch trials” (*p* < 0.05). Beta-values extracted from right posterior insula regarding the performance of either romantic or control caress are depicted in Figure 4.

**Figure 5:**
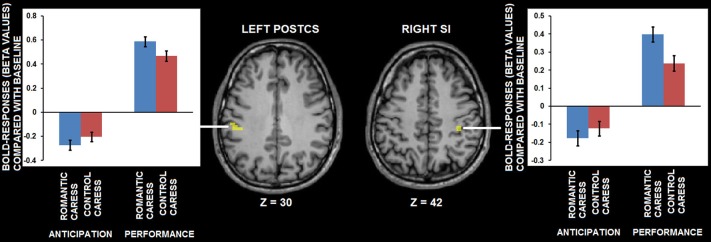
**Group statistical maps and graphs depicting differential BOLD-response for the romantic caress and control caress condition, both during caress anticipation and during caress performance, as detected by conjunction analysis thresholded at *t* > 3.61, *p* < 0.01 corrected.** Abbreviations: PostCS: postcentral sulcus; SI: primary somatosensory cortex.

A further, explorative, analysis concerning both the “no touch trials” and the “touch trials” based on conjunction analysis showed that left PostCS and right SI were differentially modulated by a romantic caress, compared to a control caress, not only during their anticipation, but also during their performance. In particular, both regions were characterized by weaker activity (i.e., stronger negative BOLD-response) during the anticipation of a romantic caress, compared to a control caress, whereas they showed stronger activity (i.e., stronger positive BOLD response) during the performance of a romantic caress, compared to a control caress (*t* > 3.61; *p* < 0.01 corrected). Statistical information about the conjunction analysis results is provided in Table 1. Group statistical maps and graphs regarding the conjunction analysis are depicted in Figure 5.

### functional magnetic resonance imaging (fMRI) functional connectivity analysis

Functional connectivity analysis demonstrated that right posterior insula significantly interacted with bilateral mid-posterior cingulate cortex, bilateral pre- and postcentral gyrus (PostCG), bilateral parietal operculum, bilateral FG, bilateral thalamus, right lateral occipital-temporal junction and right globus pallidus (GP) (*t* > 5.22; *p* < 0.0001, corrected).

Functional connectivity analysis with PLS scores as covariate showed that functional interactions between right posterior insula and right parahippocampal cortex (cluster peak coordinates: 32, −29, −15; cluster size = 351) significantly correlated with the desire for union with the other (*r* > 0.55, *p* = 0.005, corrected).

**Figure 6:**
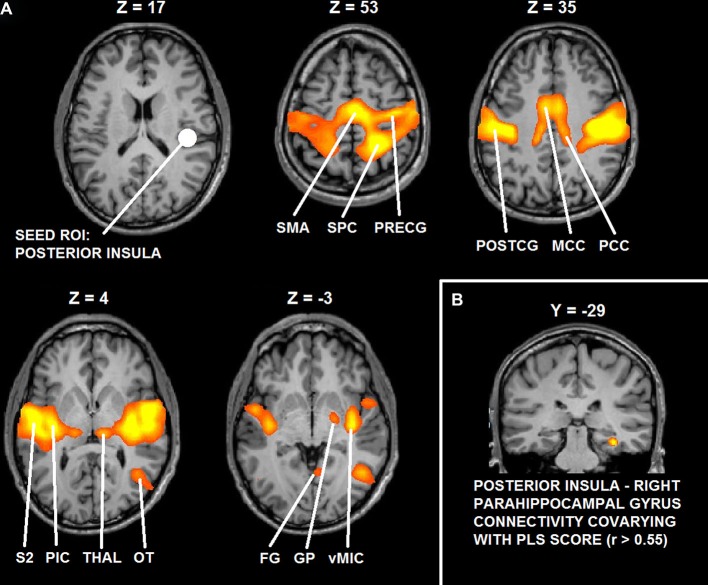
**(A)** Functional connectivity maps of the seed ROI in right posterior insula. **(B)** Group statistical maps of posterior insula connectivity covarying with PLS scores in right parahippocampal cortex. Abbreviations: SMA: supplementary motor area; SPC: superior parietal cortex; PreCG: precentral gyrus; PostCG: postcentral gyrus; MCC: mid cingulate cortex; PCC: posterior cingulate cortex; S2: secondary somatosensory cortex; pIC: posterior insula; Thal: thalamus; OT: occipital-temporal cortex; FG: fusiform gyrus; GP: globus pallidus; vMIC: ventral mid insular cortex.

Figure 6 depicts the results of functional connectivity analysis (**A**: group statistical maps of posterior insula connectivity, and **B**: correlation between posterior insula-parahippocampal connectivity and PLS scores).

## Discussion

The present study aimed at investigating the neural correlates of the desire for romantic caress by means of fMRI scanning. An fMRI-compatible experimental paradigm was applied in order to isolate the specific anticipatory representations of romantic caress (intending to caress another human being), compared with control caress (intending to caress an object). Specifically, we investigated the relationship between anticipatory neural activity and measures of feelings of passionate love for the other, that is, the intensity of the desire for union with the other. It was hypothesized that BOLD-response in anticipation of active romantic caress, compared with active control caress, in brain regions associated with the processing of affective and reward information of touch co-varied with the intensity of the desire for union with the other. Functional connectivity analysis was subsequently applied in order to gain more insight in the functional interactions in terms of long-range communication of brain regions that could be associated with the desire for romantic caress.

Summarizing the main results, whole-brain, voxel-wise analysis showed that BOLD-response in right posterior insula in anticipation of romantically caressing one’s partner, compared with an object, significantly co-varied with PLS scores, that is, the desire for union with the other. Furthermore, although pleasantness may be considered part of passionate love, this correlation between anticipatory activity in posterior insula and PLS score was independent from pleasantness ratings. Although posterior insula activity at the average group level did not significantly differ between the anticipation of romantic caress performance and the anticipation of control caress, additional ROI analysis indicated that posterior insula activity significantly differentiated between the actual performance of a romantic and a control caress, being increased for the former, compared with the latter. As evidenced by covariance analysis, the difference in posterior insula activity between the anticipation of romantic caress performance and control caress performance depended on how the participant felt for the other rather than on what was going to be touched *per se*. Connectivity analysis revealed that right posterior insula was embedded in a functional network including regions associated with reward processing and anticipation (e.g., Elliott et al., 2000; Schultz, 2000) and, consistent with previous functional connectivity studies, sensory-motor circuits (Taylor et al., 2009; Kurth et al., 2010; Deen et al., 2011; Ebisch et al., 2011; Chang et al., 2013). Moreover, functional connectivity between right posterior insula and right parahippocampal cortex positively co-varied with PLS scores.

The insula is considered a central brain structure for sensorimotor, visceral, interoceptive, homeostatic/allostatic functions, interacting with limbic, somatosensory and motor regions (Augustine, 1996; Critchley, 2005; Craig, 2009). Thalamo-cortical pathways provide a direct representation of homeostatic afferent information to posterior insula that engenders distinct bodily or interoceptive feelings by projections onto the anterior insula for subjective and conscious emotional evaluation (see also Saper, 2002). More specifically regarding the cutaneous senses, posterior insula could constitute the primary cortical locus of an interoceptive system regulating affective feelings states from the skin. Specific afferent pathways have been identified projecting threatening (Craig, 2002) or comforting (Olausson et al., 2002; Löken et al., 2009) cutaneous information via thalamic nuclei to posterior insula.

Beyond these merely perceptual functions, the insular cortex contributes to active behavior, too. For example, intracortical electrical microstimulation of the macaque insular cortex evoked a variety of emotional as well as non-emotional behaviors (Jezzini et al., 2012). In particular, stimulation of mid-posterior insula evoked affiliative and communicative behavior (Caruana et al., 2011). Based on processing of homeostatic emotions reflecting the physiological needs of the body (Craig, 2002), posterior insula also could accommodate affective/motivational functions driving behavior (Craig, 2003). For example, it has been argued that the insular cortex, integrating interoceptive information about the internal state of the body, also generates predictions about bodily feeling states (Paulus and Stein, 2006; Lovero et al., 2009) and provides a crucial neural substrate for reward-processing (Paulus, 2007). Regarding romantic relationships, insular cortex has been associated with sexual desire and love with a posterior-to-anterior pattern, implying that desire for pleasant physical contact with others could be grounded in representations of affective visceral sensations related to pleasant sensorimotor experiences and their anticipation (Cacioppo et al., 2012).

Finally, some previous studies associated posterior insula with anticipatory functions attenuating subsequent neural responses. Blakemore et al. (1999, 2000) showed decreased neural activity in S2/posterior insula for self-produced tactile stimuli on the palm of the hand, compared with externally produced tactile stimuli, suggesting attenuation of tactile perception by sensory prediction mechanisms (Blakemore et al., 1998, 1999; Carlsson et al., 2000; Hughes et al., 2013). Furthermore, Simmons et al. (2009) showed stronger deactivations in posterior insula in anticipation of either positive or negative emotional stimuli after administrating selective serotonin reuptake inhibitors to participants. These findings indicate a role of posterior insula in a bottom-up suppression of physiological arousal induced by emotional stimuli.

In accordance with this information, the present results confirm the hypothesis that posterior insula could accommodate anticipatory functions underlying affiliative behavior, and active romantic caress in particular. These findings add to the existing literature by providing further insight on how subjective factors, like the psychological relationship between individuals, mediate between predictive brain function and social interaction, that is, how anticipatory processes may shape social behavior and the experience of social interaction.

However, what would such anticipatory activation patterns in posterior insula functionally entail? It is already known that one of the consequences of such anticipatory processing is the modulation of neural responses to sensory input, either suppressing or amplifying experience (Hughes et al., 2013; van Ede et al., 2013). A closer look at the fMRI group statistical maps of the present study reveals that posterior insula was characterized by negative BOLD-responses in anticipation of touch performance, compared with baseline, whereas it was positively modulated during touch performance. Similar patterns could be observed in somatosensory cortices, especially left PostCS and right SI. Whereas a stronger deactivation (negative BOLD regulation) was detected in somatosensory cortices during the anticipation of active romantic caress, compared to control caress, during performance neural activity was stronger for romantic caress, compared to control caress in these regions. In addition, the results from co-variance analysis suggest that higher PLS scores of participants were accompanied by a weaker deactivation in posterior insula in anticipation of romantic caress, compared with the anticipation of control caress. Hence, based on our, and previous (Blakemore et al., 2000; Carlsson et al., 2000; Simmons et al., 2009; Schafer et al., 2012) results, we propose that suppression of physiological arousal in posterior insula in anticipation of active romantic caress could be modulated as a function of the desire to experience that touch; the stronger the desire, the weaker the suppression (and, in turn, the stronger the affective intensity of touch experience).

Some previous studies demonstrated a direct link between reward and somatosensation suggesting that reward anticipation modulates tactile processing (Pleger et al., 2008, 2009). One may hypothesize that also posterior insula contributes to such effects in the context of an affective, romantic caress. Interestingly, although left PostCS and right SI, like posterior insula, differentiated between a romantic and a control caress during performance, unlike posterior insula, left PostCS and right SI activity did not co-vary with PLS scores. This suggests that SI/PostCS activity patterns may be related to distinct aspects of romantic caress processing, possibly based on interoceptive and exteroceptive/proprioceptive functions generally attributed to posterior insula (Craig, 2002) and postcentral somatosensory cortices (Keysers et al., 2010), respectively. Further studies would be necessary to confirm this hypothesis more directly, and to investigate more in detail the relationship between anticipatory activation patterns and subsequent stimulus processing as well as the neurophysiological or psychological arousal induced by the stimulus, and possibly distinguishable roles of postcentral somatosensory cortices and posterior insula.

Finally, functional connectivity analysis, measured as temporally-correlated patterns brain activity across brain regions reflecting an index of brain long-range communication (Fox and Raichle, 2007; Van Dijk et al., 2010), suggests that other brain regions involved in reward anticipation (Elliott et al., 2000; Schultz, 2000) may support the role of posterior insula in anticipation of romantic caress, i.e., globus pallidus (GP), and its modulation by the desire for that touch, i.e., parahippocampal areas. Indeed, GP was functionally linked to feelings of love for another (Acevedo et al., 2012) and is anatomically connected with posterior insula via the ventral striatum (Chikama et al., 1997) also involved in associating tactile stimuli with reward (Pleger et al., 2008, 2009). Moreover, previous studies showed that parahippocampal repetition suppression is sensitive to reward-predicting properties of stimuli (Zweynert et al., 2011). Reward-predicting properties of stimuli have been also shown to enhance activity in the hippocampus, in addition to recollection of the same stimuli (Wittmann et al., 2005). Thus, it has been proposed that reward prediction might enhance hippocampus-dependent memory formation (Wittmann et al., 2005). Although it needs to be recognized that the present results do not provide direct evidence for involvement of reward circuitry, based on this knowledge it is possible to speculate that the modulation of posterior insula activity in anticipation of romantic caress by the desire for that touch might rely on reward-dependent memory of romantic caress experience.

Some other issues need to be mentioned. First, previous studies already investigated the neural correlates of pleasant and romantic caress experiences. How do they relate to or differ from the present study? Regarding the neural substrates of the emotional aspects of pleasant touch experiences, Rolls et al. (2003) showed that certain areas of the human orbitofrontal cortex and cingulate cortex are involved in representing pleasant touch. Other studies demonstrated coding of pleasant romantic caress in posterior insula (Morrison et al., 2011) based on the C-tactile fiber system projecting to posterior insula (Olausson et al., 2002; Löken et al., 2009). Additional experiments showed that the representation of pleasant romantic caress also involves right posterior superior temporal sulcus and the medial prefrontal cortex/dorsal anterior cingulate cortex (Gordon et al., 2013) and that anterior insula is involved in the anticipation of the sensory-affective impact of touch, predicting BOLD-responses during subsequent passive touch experiences in posterior insula (Lovero et al., 2009). Finally, Gazzola et al. (2012) reported activation in SI being associated with the affective processing of passively experienced romantic caress as well as its prediction. This neural activation also was modulated by the perceived gender of the other individual. Whereas we found significant modulations of BOLD-response in posterior insula, these where not detected in other brain regions associated with pleasant touch experiences. However, some relevant differences between studies can be noted.

Beside the difference with these previous studies in terms of active and passive touch, another important difference is that, here, we specifically focused on the psychological relationship between individuals (i.e., intensity of feelings of passion for the other), whereas previous studies investigated the affective perceptual qualities of the touch, like its pleasantness. Although pleasantness may be considered part of passionate feelings, results suggest that desire and pleasantness are not necessarily interdependent and reflect, at least partially, distinct phenomena. First, although there was a trend towards significance, we failed to detect a significant statistical dependency between them. Second, co-variance of posterior insula activity with PLS scores appeared independent of pleasantness ratings, suggesting that anticipatory activity for active romantic caress in posterior insula specifically depends on the intensity of longing for the other, but not on the sensory-affective qualities of the touch itself. In this sense, the present results suggest a mechanism for the desire for romantic caress that is distinct from or goes beyond the anticipation of the sensory-affective qualities of the touch.

However, it should be noted that previous studies also reported coding of the affective qualities of perceived romantic caress, like its pleasantness, in posterior insula (e.g., Morrison et al., 2011). A relevant difference with these studies is that they concerned touch on the back of the hand (C-tactile innervated skin) stimulating the C-tactile fiber system projecting to posterior insula (Olausson et al., 2002; Löken et al., 2009), whereas, here, participants caressed another individual with their fingers and palm of the hand (glabrous skin). Anticipation in posterior insula/parietal operculum based on sensory prediction mechanisms of tactile stimuli on the palm of the hand also has been reported (Blakemore et al., 1999, 2000). Future experiments would be needed to address this apparent discrepancy. Likely, different neuro-functional mechanisms within posterior insula are involved in processing desire and sensory-affective information of romantic caress, based on its interoceptive (Craig, 2003) and (exteroceptive) sensory-affective (Olausson et al., 2002) functions. A relevant detail supporting this explanation is that the association between anticipatory activity and PLS scores was detected in right posterior insula, that is, ipsilaterally to the touching hand. Moreover, differential activity was detected in the same cluster during the actual performance of a romantic caress, compared to a control caress. These findings further suggest that the detected association may not be directly related to the anticipation of basic sensory-affective experiences, more likely to involve contralateral posterior insula. Indeed, insula cortex is involved in manifold functions, including the processing of sensory, affective and bodily information about its physiological state and needs, at the basis of its integrative and social properties (Craig, 2009; Singer et al., 2009).

Second, the present study included romantically involved couples. It could be argued that a second group of participants touching unknown individuals might have provided relevant additional information. Extending the current results, in that case, a stronger deactivation in posterior insula in anticipation of a romantic caress could be expected, being the most arousing condition due to its social valence, considering that there possibly is less desire to experience that arousal in unrelated individuals. However, one also may argue that it would be difficult to isolate the desire component from other confounding factors when comparing groups, while it was not when studying variable degrees of desire within romantically involved participants. Nevertheless, we propose that it would be relevant for future work to compare different groups of interacting participants in order to investigate the influence of specific relationships between individuals.

In conclusion, the present results show that neural activity in posterior insula in anticipation of the performance of a romantic caress varies as a function of the desire for union with the other. The findings suggest that posterior insula, interacting with brain regions related by previous studies to sensory-motor and reward functions, could provide a neural substrate mediating between the desire for tactile contact with others and active romantic caress. In particular, anticipatory activity patterns in posterior insula possibly modulate subsequent affective processing of skin-to-skin contact.

## Author contributions

Sjoerd J. Ebisch, Francesca Ferri and Vittorio Gallese designed research; Sjoerd J. Ebisch and Francesca Ferri performed research; Sjoerd J. Ebisch analyzed data; Sjoerd J. Ebisch, Francesca Ferri and Vittorio Gallese wrote the paper.

## Conflict of interest statement

The authors declare that the research was conducted in the absence of any commercial or financial relationships that could be construed as a potential conflict of interest.
